# Destructive and Non-Destructive Testing of the Performance of Copper Slag Fiber-Reinforced Concrete

**DOI:** 10.3390/ma15134536

**Published:** 2022-06-28

**Authors:** Vijayaprabha Chakrawarthi, Brindha Dharmar, Siva Avudaiappan, Mugahed Amran, Erick Saavedra Flores, Mohammad Ayaz Alam, Roman Fediuk, Nikolai Ivanovich Vatin, Raizal S. M. Rashid

**Affiliations:** 1 Department of Civil Engineering, Alagappa Chettiar Government College of Engineering and Technology, Karaikudi 630003, India; vijayaprabha.struct@gmail.com; 2 Department of Civil Engineering, Thiagarajar College of Engineering, Madurai 625015, India; dbciv@tce.edu; 3 Departamento de Ingeniería Civil, Universidad de Concepción, Concepción 4030000, Chile; 4 Department of Civil Engineering, College of Engineering, Prince Sattam Bin Abdulaziz University, Alkharj 16273, Saudi Arabia; 5 Department of Civil Engineering, Faculty of Engineering and IT, Amran University, Amran 9677, Yemen; 6 Departamento de Ingeniería en Obras Civiles, University of Santiago of Chile, Av. Ecuador 3659, Santiago 9170201, Chile; erick.saavedra@usach.cl; 7 Departamento de Geología, Facultad de Ingeniería, Universidad de Atacama, Avenida Copayapu 485, Copiapó 1531772, Región de Atacama, Chile; ayaz.alam@uda.cl; 8 Polytechnic Institute, Far Eastern Federal University, 690922 Vladivostok, Russia; fedyuk.rs@dvfu.ru; 9 Peter the Great St. Petersburg Polytechnic University, 195251 St. Petersburg, Russia; vatin@mail.ru; 10 Department of Civil Engineering, Faculty of Engineering, Universiti Putra Malaysia, Serdang 43400, Malaysia; raizal@upm.edu.my

**Keywords:** copper slag, polypropylene fiber, NDT, regression analysis

## Abstract

Concrete technology is adopted worldwide in construction due to its effectiveness, performance, and price benefits. Subsequently, it needs to be an eco-friendly, sustainable, and energy-efficient material. This is achieved by replacing or adding energy-efficient concrete materials from industries, such as ground granulated blast furnace slag, steel slag, fly ash, bottom ash, rice husk ash, etc. Likewise, copper slag is a waste material produced as molten slag from the copper industry, which can be used in concrete production. Copper slag can perform roles similar to pozzolans in the hydration process. This paper extends the comparative study of copper slag concrete with polypropylene fiber (PPF) subjected to destructive and non-destructive testing. Under destructive testing, compressive strength of concrete cubes, compressive strength of mortar cubes, splitting tensile tests on cylindrical specimens, and flexural tests on plain cement concrete were conducted and analysed. Ultrasonic pulse velocity and rebound hammer tests were performed on the samples as per IS13311-Part 1-1992 for non-destructive testing. The 100% replacement of copper slag exhibited a very high workability of 105 mm, while the addition of 0.8% PPF decreased the flowability of the concrete. Hence, the workability of concrete decreases as the fiber content increases. The density of the concrete was found to be increased in the range of 5% to 10%. Furthermore, it was found that, for all volume fractions of fiber, there was no reduction in compressive strength of up to 80% of copper slag concrete compared to control concrete. The 40% copper slag concrete was the best mix proportion for increasing compressive strength. However, for cement mortar applications, 80% copper slag is recommended. The findings of non-destructive testing show that, except for 100% copper slag, all mixes were of good quality compared to other mixes. Linear relationships were developed to predict compressive strength from UPV and rebound hammer test values. This relationship shows better prediction among dependent and independent values. It is concluded that copper slag has a pozzolanic composition, and is compatible with PPF, resulting in good mechanical characteristics.

## 1. Introduction

Concrete is a versatile material, popular for its low cost and adaptability [1]. It is the second-largest material consumed by humanity, second only to water [2]. Concrete is a composite paste of calcareous and argillaceous material, such as cement, sand, and coarse aggregate, mixed with water [3]. A material other than cement water, known as an aggregate, provides high dimensional stability to the concrete, since 55–80% of the concrete volume consists of aggregates. It also determines the particle packing and strength of the concrete. Each material contributes a unique property to develop a homogeneous mass. The total construction cost depends on the number of individual constituents. Among these materials, sand and aggregates are the non-renewable resources under the highest demand among construction industries [4]. The availability of sand is diminishing, and its cost is increasing daily [5,6]. 

Natural resources are diminishing at their source due to recent development in urbanization and industrialization. Sand is one of such natural resources disappearing daily due to large mining operations utilized for construction [7]. The illegal mining of river sand near the river bed affects the surrounding ecology; aquatic mining also leads to soil erosion, intrusion of seawater, and water contamination, etc. Altogether, some of the industrial wastes disposed of into open land cause environmental pollution and contaminate nearby water bodies, leading to severe health hazards and loss of aquatic life [8]. Industrial waste faces problems in terms of disposal methods, increasing costs of disposal, health hazard issues in metal recovery and water treatment industries, etc., and needs sustainable decisions for the safe and productive disposal of waste [7,9]. Industrial wastes have advantageous components, such as silica, calcium oxide, ferrous metals, etc., which can produce harder mass when incorporated into concrete [10]. As a solution to river sand scarcity and industrial waste recycling, such wastes can be substituted into cement, aggregate, or both, resulting in sustainable concrete and green technology [11,12,13,14,15].

Researchers are interested in replacing bottom ash, blast furnace slag [1], copper slag, steel slag, furnace slag, iron scales, ceramic waste, e-waste, marble dust, and recycled aggregate in the place of river sand in concrete [4,5,7,8,9,10,11,12]. Reportedly, slag is the 16th element used in creating high-performance concrete [16], and is known as a supplemental cementitious material (SCM) [17], which can improve the strength and durability of concrete [18]. With a surface area of 400–600 m^2^/kg and a bulk density of 1200 kg/m^3^, fine slag powder may be combined with free lime to replace 40–65% of total cement content. On the other hand, the coarser slag powder can be employed as an aggregate substitute in the concrete matrix [19]. Despite its low hydration activity, the slag has cementitious properties similar to ordinary Portland cement (OPC) [20,21,22,23,24]. As a result, slag has several advantages in manufacturing concrete [20,21,22,23]. It decreases cement hydration heat, improves long-term compressive strength, increases durability, and considerably reduces the adiabatic temperature rise in concrete [24,25]. Ground granulated blast furnace slag is one form of slag that can be used to replace the high cement content in concrete [26]. However, it may have lower mechanical strength [27].

Furthermore, because of the large particle size of slag, it has weaker cementing characteristics, which may decrease the bond between slag particles and calcium–silicate–hydrate gels [28,29]. Under significant volumes of steel slag, coarser pore structures can emerge [30], reducing concrete strength and durability [31]. However, earlier research has found that a slag-based composite binder can minimize setting time, enhance concrete rheology, refine porosity, and provide sufficient compressive strength [32,33]. In a previous study, the composite binder displayed decreased hydration heat, and the concrete adiabatic temperature rose compared to another common additive—flying ash [32]. The GGBFS exhibits a good pozzolanic response, absorbing Ca(OH)_2_ to react with sulfate and reducing gypsum [34]. Due to the development of C–S–H gels via a pozzolanic reaction, the resulting slag concrete is denser and has better sulfate resistance [35].

Copper slag is a waste derivative obtained from the smelting and refining process during copper separation from copper ore [1]. Using copper slag in cement and concrete enhances the quality of concrete, providing an environmentally sustainable material with cost-effectiveness [36]. Copper slag is widely used in the sandblasting industry and in manufacturing abrasive tools, such as cutting, polishing, and grinding tools [37]. Many works in the literature testify that copper slag is one of the alternates to fine aggregates, and can also possibly produce good concrete. Copper slag holds a high specific gravity; hence, the concrete is 5% denser than ordinary concrete [38]. 

Moreover, copper slag has an irregular glassy texture that does not absorb large quantities of water [39,40]. Therefore, the water absorption rate in copper slag is meager compared to river sand. In addition, its irregular texture results in sharp edges that provide better bonding between aggregate cement matrixes, and thus make a more robust interfacial zone [41]. Therefore, the workability of concrete improves due to the lower absorption rate of copper slag. However, in the case of more extensive substitution of copper slag, the lower water absorption rate affects the performance of fresh concrete properties [40]. Reportedly, 80–100% replacement lead to aggregate settlement and the floating of excess water, resulting in bleeding and segregation [42,43]. 

The effect of copper slag on concrete strength after a 56- and 90-day curing period evidenced higher concrete strength, and in no case was the strength reduced. Additionally, each mix has a variation of high strength values compared to ordinary concrete [44,45]. The graph profile shows a gentle slope of increasing compressive strength up to 50% copper slag, and, in the case of tensile strength, up to 40% copper slag replacement [46]. The sharp and irregular texture ensures better particle packing under pressure, reducing the stress at critical points. The voids are filled with water, and the cement–copper slag matrix reduces voids by up to 40% [47,48]. A combination of cement, copper slag, and water-produced concrete mortars incorporating copper slag exhibit 1.14 times higher compressive strength [40]. ASTM fixed criteria for a pozzolan requires that the total percentage of silica, alumina, and ferric oxide should be more than 70%. In the case of copper slag, it was found to be around 95%. Therefore, copper slag is proven to be a high-quality pozzolanic material. The heavy metal leaching was analyzed, and all heavy metals were under safe limits, even in extremely hostile situations. Some studies have included 2% hydraulic lime to accelerate the pozzolanic reaction [49,50,51].

Slag concrete was also found to show better durability and higher corrosion resistance [51]. The ferric oxide content in copper slag makes the concrete susceptible to acid attack [52,53]. At the same time, copper slag concrete shows superior performance in protecting the concrete against sulfate attack in the case of fine aggregate replacement, wherein the resistance is comparatively low when copper slag is replaced by cement [51]. Polypropylene fiber is added to enhance flexural, tensile, toughness, and impact properties [54,55,56]. PPFs above 0.2% in volume show a reduction in workability [57,58], and fiber content beyond 1% reduces the strength [59,60]. Use of staple or fibrillated fiber instead of monofilament fiber showed increased compressive strength (14.60–17.31%), splitting tensile strength (8.88–13.35%), and modulus of rupture (8.99–24.60%), and decreased shrinkage stain (0.862 to 0.871) [61,62,63]. Very few researchers to date have concentrated on fully replacing copper slag in concrete and cement mortar applications. Moreover, none of the previous studies considered copper slag concrete combined with PPF. Hence, this experimental work concentrates on the assessment of workability parameters, density variation of concrete, compressive strength of concrete, mortar compressive strength, and non-destructive evaluation of different replacements of copper slag and polypropylene fiber. Copper slag replacement levels are 20%, 40%, 60%, 80%, and 100% regarding river sand, whereas the PPF volume fraction ranges are 0.2%, 0.4%, 0.6%, and 0.8%.

## 2. Materials and Methods

### 2.1. Materials

**Figure 1:**
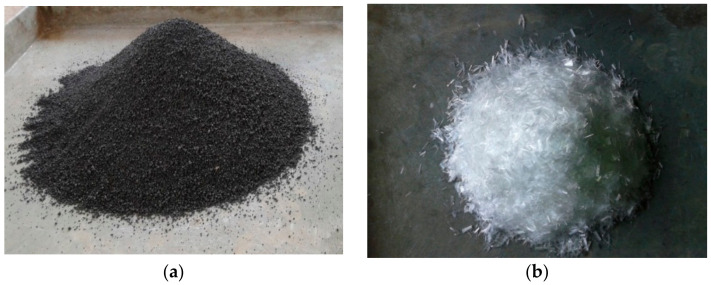
(**a**) Copper slag and (**b**) PPFs.

Ordinary Portland cement (OPC, 43 grade conforming to IS 8112-2013 [64]) was used throughout this study. Crushed stone aggregate of 20 mm maximum size, was used as coarse aggregate. River sand was combined with a suitable proportion of copper slag purchased from the Sterlite Corporation of India, Tuticorin. Drinking water is used for concreting works. Naphthalene-based superplasticizer Conplast SP430 was used. The dosage was fixed as 0.5% of the weight of cement. Fibrillated polypropylene fiber of 0.91 kg/m^3^ density was obtained from Jeetmull Pvt Ltd., Chennai, India. Figure 1a,b shows images of the copper slag and fibrillated polypropylene fiber. Aggregates were tested conforming to IS 2386-1963 [65] codal provisions; the tabulated physical properties are presented in Table 1.

From the obtained results of fundamental properties of copper slag, it was noted that the water absorption was 88% lower than for the sand. When copper slag is replaced at a higher rate, the unoccupied moisture content accumulates [66]. Surplus concrete water accelerates the hydration process, and bleeds in 100% replacement. The chemical composition of cement, copper slag, sand, and PPF are compared in Table 2. The chemical compositions of various ingredients were obtained using the XRD technique.

### 2.2. Gradation of Fine Aggregate

**Figure 2:**
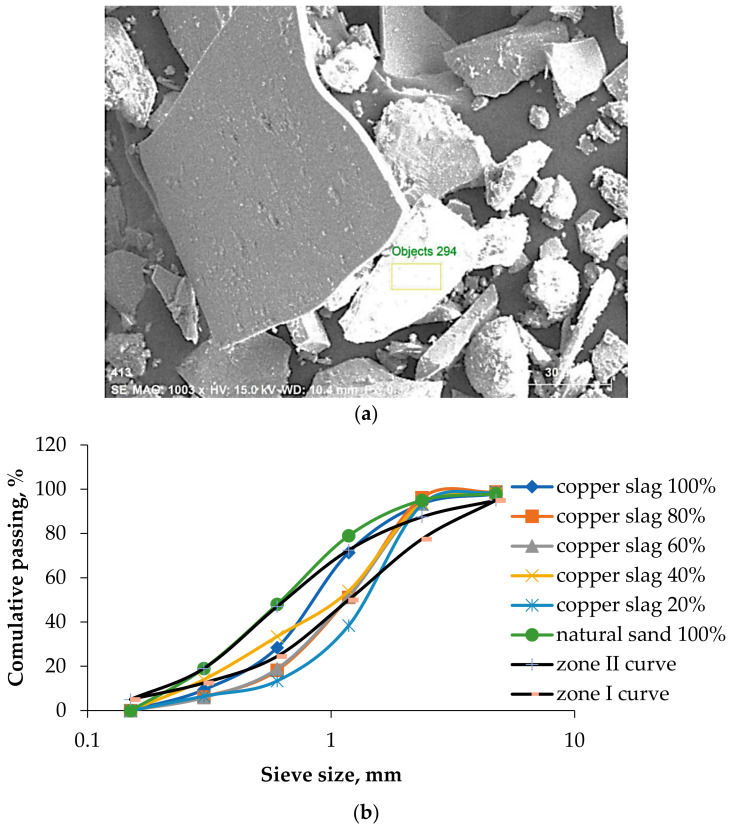
(**a**) SEM image of copper slag and (**b**) grading curves of various copper slag proportion.

Copper slag particles are available from 4.75 mm to 0.075 mm in size and exhibit a proper grading and good particle packing. Figure 2a shows the particle size of copper slag on a 30 µ scale. Copper slag constitutes a 90% high-stability glassy material that does not absorb or allow water to penetrate. This property attributes the prevention of moisture intrusion inside the concrete, providing long life to the structure. Since copper slag is a high specific gravity material, the equivalent weight of copper slag to the quantum weight of natural sand is higher. In addition, the particles are densely packed among cement particles and coarse aggregates in concrete. Based on sieve analysis, 40% and 100% copper slag have the same percentage passing as sand. All proportions come under zones II and III of the Indian standard classification [67,68]. From the semi-log graph shown in Figure 2b, it is clear that 20% and 60% are deviating more from the natural sand. From these results, it was concluded that copper slag can be used as a fine aggregate without any alteration in grading.

### 2.3. Concrete Proportioning

Concrete proportioning was conducted on copper slag replacement from 0% to 100% and fibrillated polypropylene fiber from 0% to 0.8%, in increments of 20% and 0.2%, respectively. Cement mortars were cast on a 1:3 ratio, in which the sand was replaced up to 100%. The cement content was taken as 360 kg/m^3^, and the water–cement ratio was fixed as 0.41. The mix proportions and specimen details for various testing are listed in Table 3 and Table 4, respectively.

## 3. Experimental Procedures

### 3.1. Destructive Testing

**Figure 3:**
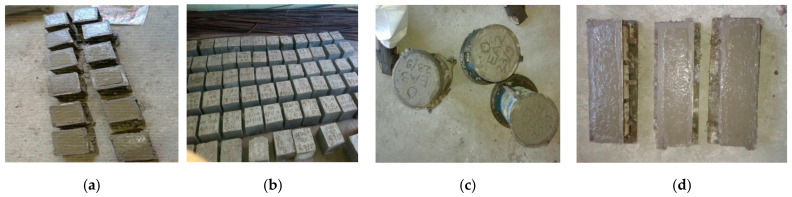
Cast of specimens processed: (**a**) cube samples, (**b**) samples dismantled from the molds, (**c**) cylinder samples, and (**d**) prism samples.

**Figure 4:**
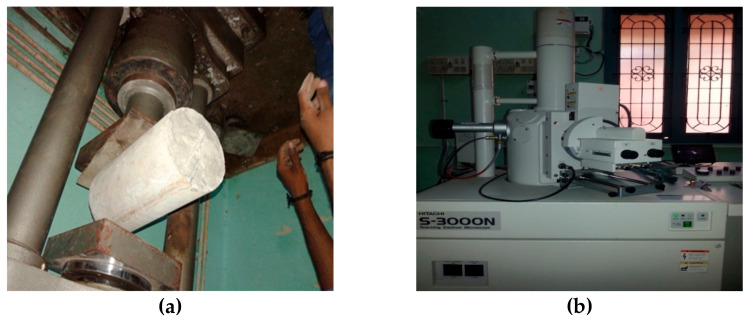
(**a**) Compression testing machine and (**b**) scanning electron microscope.

The slump and compacting tests were performed according to Indian standard IS-1199-1959 [69] to analyze the workability characteristics. The dry weight of the concrete cubes was noted to find the dry density of concrete before testing the compression test. Compressive strength tests on concrete cubes and cement mortar cubes were conducted on cubes of sizes 150 mm × 150 mm × 150 mm and 70.6 mm × 70.6 mm × 70.6 mm, respectively. A compression testing machine set at 2000 kN and 300 kN was used to conduct the compression test on concrete and mortar cubes. The test was performed according to the codal provision IS 516-1959 [70]. Figure 3 shows cast specimens for different destructive tests. The splitting tensile strength is determined at 28 days on cylinders of 150 mm in diameter and 300 mm in length, as per IS: 516-1959. Microstructural images are captured via HITACHI-3000N scanning electron microscope. Figure 4 illustrates the split tensile test using the compression testing machine (Figure 4a) and scanning electron microscope (Figure 4b). An array of electrons focused on the sample and backscattered the secondary electrons. The signals detect the secondary electrons to produce the nanoscale images. 

### 3.2. Non-Destructive Testing

#### 3.2.1. Ultrasonic Pulse Velocity

**Figure 5:**
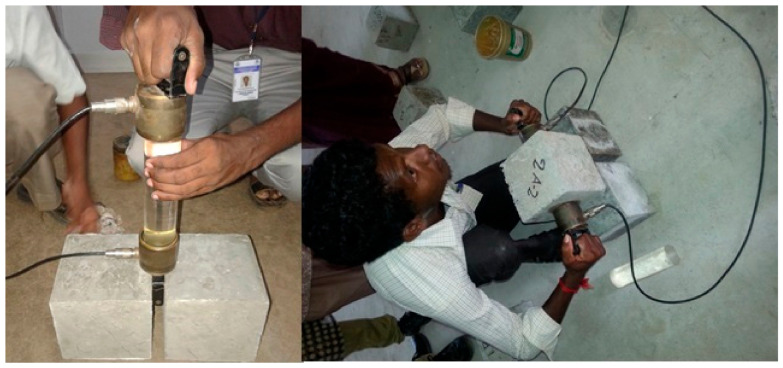
Test set up for ultrasonic pulse velocity test.

The UPV test is an in situ, qualitative, non-destructive testing methodology for concrete conducted per the procedure in IS: 13311:1992 [71]. Before conducting destructive tests, this test was performed on 150 mm × 150 mm × 150 mm cubes. The UPV test is used to analyze the porous, cavities, and non-homogeneity of the concrete in terms of applying and receiving the electronic pulses on both sides of the concrete specimen. The velocity of electronic waves is influenced by the concrete grade, path length, curing period, and maximum size of aggregates. The instrument consists of a transducer, receiver or pulser (two probes), and necessary display units. The sound energy produced by the pulse passes through the transducer as wave energy. Some energy is back-reflected because of cavities and flaws inside the concrete. The time taken by the pulse to penetrate the structure is measured. After calibration with a standard sample material with known properties, the transducers were placed on opposite sides of the material. A portable ultrasonic non-destructive digital indicating tester (PUNDIT) was used to test UPV, as shown in Figure 5. Pulse velocity was measured by a simple formula (1):

(1) $$Ultrasonic\;Pulse\;velocity=\frac{Path\;length}{Transit\;time,}$$

In the case of flaws and cavities, the time taken to travel for a particular path length is high, which indicates the lower velocity of sound waves, and results in poor quality of concrete. This velocity relates to the compressive strength of concrete. Table 5 presents the pulse velocity, which relates to the quality of concrete.

#### 3.2.2. Rebound Hammer Test

**Figure 6:**
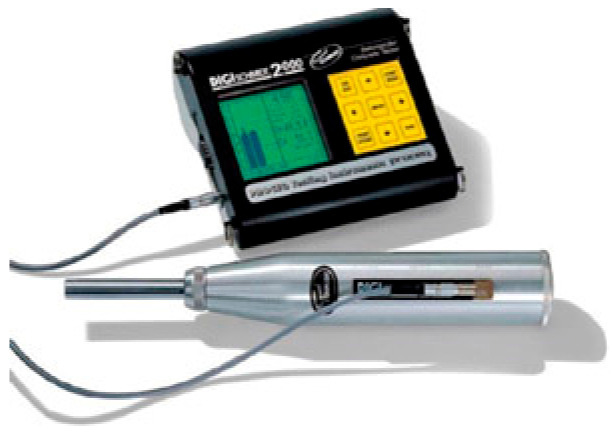
SCHMIDT rebound hammer test apparatus.

Concrete’s surface hardness is expressed as the non-destructive compressive strength of concrete. The uniformity of the concrete is affected by the compaction variability and variability in curing and temperature changes. Figure 6 illustrates the SCHMIDT rebound hammer (digital) equipment used to assess the uniformity of concrete. It was used to obtain the average compressive strength of 150 mm^3^ cube at 28 days. The surface was cleaned and free from moisture content. The impact load was applied at a constant energy of 2.2 Nm. The number of trails against each measurement was entered as input data before starting the test. The impact energy is used so that the hammer spring rebounds against the concrete surface. The surface hardness of concrete is indicated as the compressive strength. According to BIS 13311—Part 2 [71], the estimation can vary by ±25% with the experimental compressive strength results. 

## 4. Results and Discussions

### 4.1. Density

**Figure 7:**
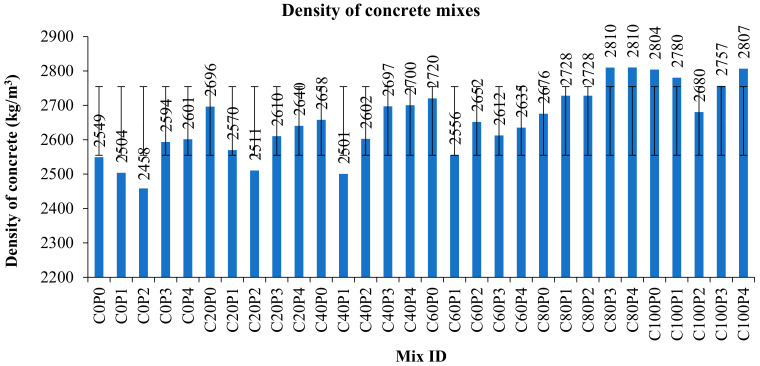
Density of various concrete mixes.

Copper slag is available in the fine aggregate state, and has a higher bulk density than river sand and crushed stone aggregate. The fineness modulus lies between sand and coarse aggregate. Copper slag occupies less volume than concrete due to its higher bulk density and specific gravity. These factors invariably influence the density of concrete. Hence, concrete with copper slag has higher density values, ranging from 2500 kg/m^3^ to 2850 kg/m^3^ [72]. A significant reduction in density was noted with the increase in fiber content, since the fiber occupies a specific volume of the concrete depending upon its percentage volume fraction in the concrete. Figure 7 shows the variation in density of all mixes. The minimum density observed was 2696 kg/m^3^, whereas the maximum density was 2810 kg/m^3^. The densities varied from 5% to 10% compared to control concrete.

### 4.2. Workability

**Figure 8:**
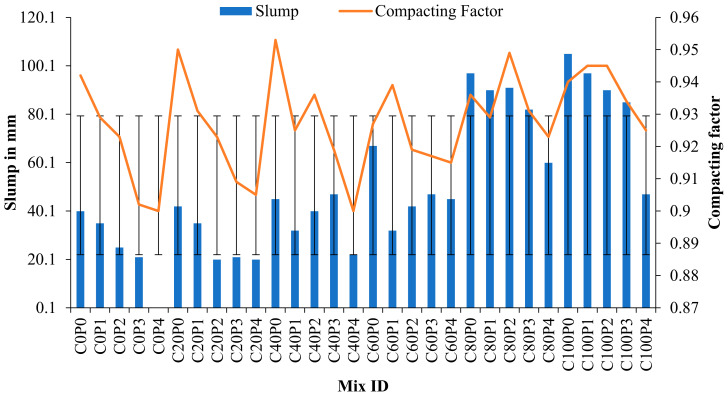
Slump and compacting factor variations.

Figure 8 illustrates the variation in the slump and compacting factors. Many studies have proven that the copper slag has outstanding workability characteristics because of its glittering texture and low water absorption capacity [48,73,74,75]. The 100% replacement of copper slag (C100P0) has a slump value of 105 mm, whereas control concrete exhibits a 40 mm slump. One study observed that 100% copper slag shows a 200 mm slump, and the control concrete exhibited 65 mm workability. However, 100% copper slag was noted to exhibit bleeding and segregation, even with the addition of PPF [37,76]. C100P4 decreases the slump value significantly to 47 mm. Adding polypropylene fiber leads to excess water demand in the interfacial transition zone (ITZ). Loss of moisture content in ITZ causes cracking. The presence of copper slag avoids fluid loss and improves the microstructure of the cement aggregate phase. Copper slag is crystalline in structure and has a glassy appearance. The surface prevents free surface moisture from entering. As a result, accessible water is conserved for hydration, which densifies the microstructure. Additionally, the diamond length of a single fibrillated polypropylene fiber is longer; hence, the cement paste needed to coat the fiber substantially reduces the fluidity of the concrete. It is worth noting that the addition of PPF maintains the ideal slump value; the sand is entirely replaced with copper slag. The concrete containing 0.8% PPF had a large lumped mass of fibers and exhibited difficulty in mixing due to the insufficient capacity of the mortar and considerable amount of fibers, which were visible in the fresh concrete mix. It was observed that clear segregation did not occur in mixes up to 80% of copper slag substitution with PPF. 

The degree of compaction was determined using a compacting factor test. The compacting factor values of copper slag–PPF concrete ranged from 0.90 to 0.95, whereas the compacting factor of control concrete was 0.942. The high specific gravity of copper slag in concrete facilitates the self-settling of aggregates, resulting in void-free concrete. Conversely, PPF added to concrete occupies a specific volume, resulting in weight loss in partially compacted concrete. Hence, there was a slight decrease in compacting factor when PPF was added to the concrete. However, this difference had no influence, since the desirable compacting factor for manually compacted heavily reinforced sections is 0.90 to 0.95. The equivalent slump values analogous to the above-mentioned compacting factors start from 50 mm to 100 mm.

### 4.3. Destructive Testing

#### 4.3.1. Compressive Strength

**Figure 9:**
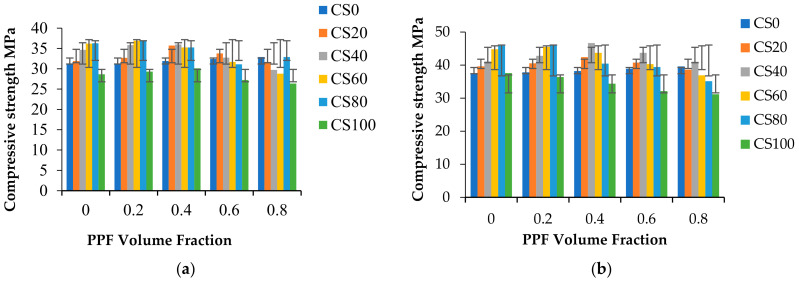

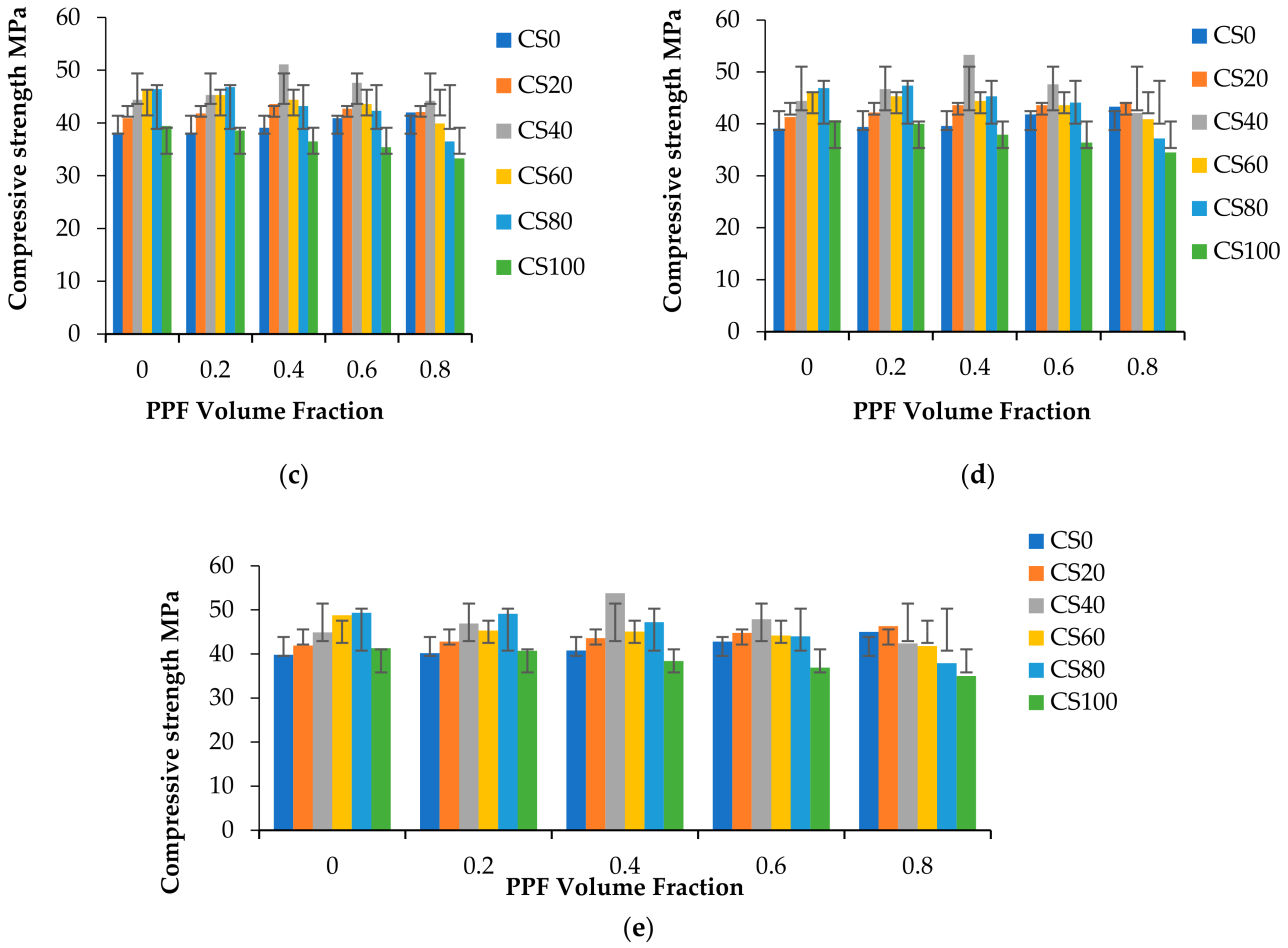
Compressive strength of concrete at 7, 28, 56, 90, and 180 days. (**a**) Compressive strength at 7 days; (**b**) Compressive strength at 28 days; (**c**) Compressive strength at 56 days; (**d**) Compressive strength at 90 days; (**e**) Compressive strength at 180 days.

From Figure 4, Figure 5, Figure 6, Figure 7, Figure 8 and Figure 9 in this study, it was predicted that strength increases as the curing period increases. Past literature [45,77,78] analyzed copper slag incorporation into high-strength concrete, cement mortar, and high-performance concrete applications. The common observation in the results of these studies was that the copper slag addition increases the compressive strength over the control concrete. Most research has concluded that 30% to 40% of copper slag is the optimum replacement [38,79,80]. Some studies have confirmed that the compressive strength gradually increases up to 60%, and then decreases.

Moreover, it was found that 100% copper slag replacement does not meet the mean target strength [38,81,82]. Adding fiber contributes to the enhancement of compressive strength by 6%. Copper slag replacement of 20% delivers a 35% improvement in compressive strength at 28 days. However, Hsie et al. [61] reported only a 14.6 to 17.35 % strength gain over control concrete. Copper slag content increases the volume of free water in the concrete. PPF contributes higher water absorption characteristics in up to 40% copper slag mixes. Beyond this limit, the surplus water remains unused by the fiber matrix regarding pronounced segregation [83]. High compressive strength was observed at 40% copper slag, and PPF 0.4% exhibited a 15 to 35% increase in strength. Since particle size distribution plays a significant role, the particle packing of 40% copper slag can maintain good characteristic strength. Copper slag 40% replacement exhibits the optimum compressive strength value of 53.8 N/mm^2^ at 180 days. PPF 0.6% can be used with 60% copper slag to produce workable concrete. After that, compressive strength decreases as copper slag replacement increases from 60% to 100%. The decrease in the compressive strength of concrete is due to the presence of freely available water in cement, but the strength value does not fall below the target mean strength or the compressive strength of control concrete. Replacement of 80% copper slag (Figure 9) achieved the highest strength (50.3 N/mm^2^) at 180 days without fiber content. The 100% replacement of copper slag to fine aggregate exhibited higher strength than control concrete. 

#### 4.3.2. Compressive Strength of Cement Mortar

**Figure 10:**
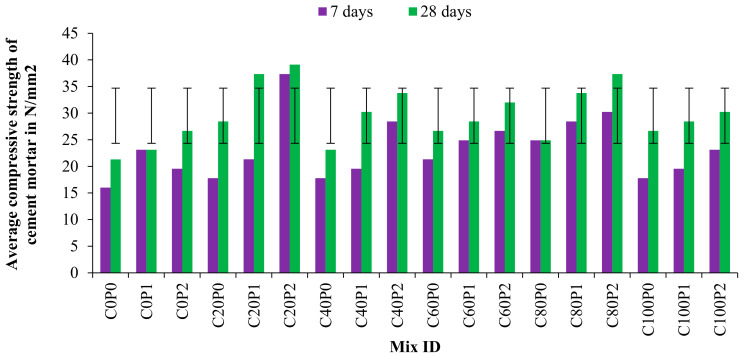
Compressive strength of cement mortar cubes.

**Figure 11:**
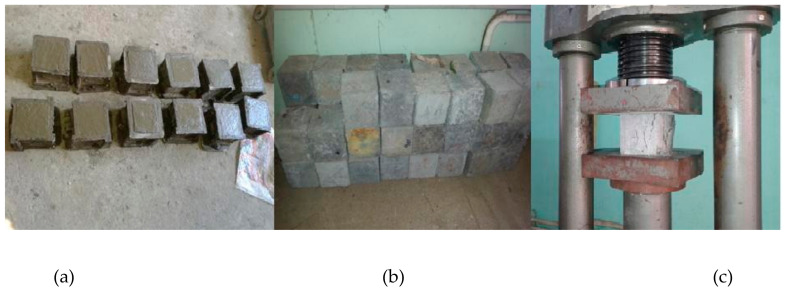
(**a**) Casting, (**b**) curing and (**c**) testing of mortar cubes.

Compressive strength results were obtained by casting and testing cement mortar cubes of size 70.6 mm × 70.6 mm × 70.6 mm at 7 and 28 days. In addition, various proportions of copper slag and polypropylene fiber constituting 180 specimens were tested; the results are tabulated in Table 6, followed by the graphical representation in Figure 10. Additionally, Figure 11 shows the sample of specimens involved in casting (Figure 11a), curing (Figure 11b) and testing (Figure 11c). 

After 7 days, the C100P2 mix shows 44% higher compressive strength. Even when the sand was 100% replaced with copper slag, it showed 11% higher compressive strength than control concrete. Compared to control concrete, the C20P2 mortar mix exhibited a 133.31% increase in compressive strength after 7 days. This was due to the confinement of the mortar mix due to the PPF in the concrete. 

The compressive strengths of cement mortar at 28 days for C20P0 mix and C80P2 mix were 39.11 N/mm^2^ and 37.33 N/mm^2^, respectively. This implies that the replacement of 20% and 80% copper slag produces 83% and 75% higher strength values than the reference mix. Copper slag replacement without PPF increased compressive strength by 11.13%, whereas the addition of PPF further improved the compressive strength by 25.57%. By no means did the compressive strength of any of the mixes fall below that of the control mix. Hence, the copper slag can be entirely replaced with sand in cement mortar application to maximize waste utilization. Table 7 illustrates the splitting and tensile strength of concrete. The ratio between splitting tensile strength and compressive strength indicates that the compressive strength is closely related to tensile stress.

### 4.4. Non-Destructive Testing

#### 4.4.1. Ultrasonic Pulse Velocity

**Figure 12:**
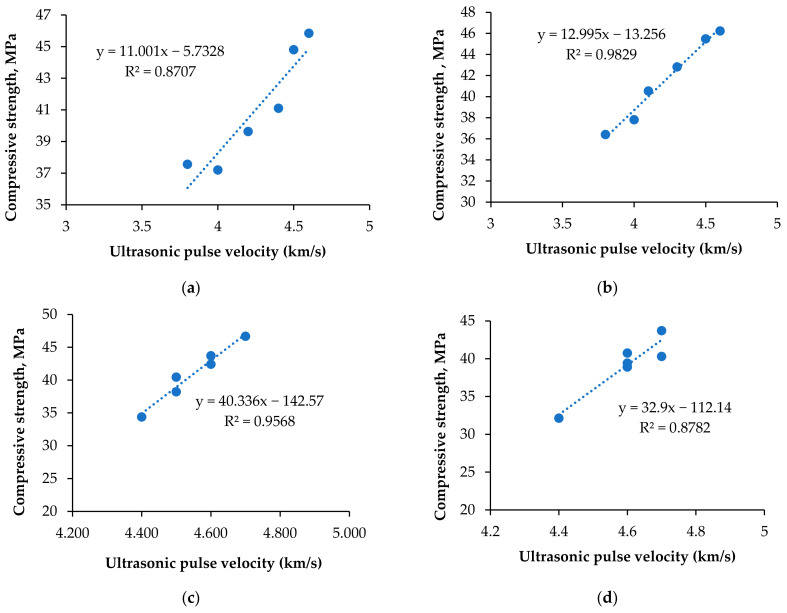

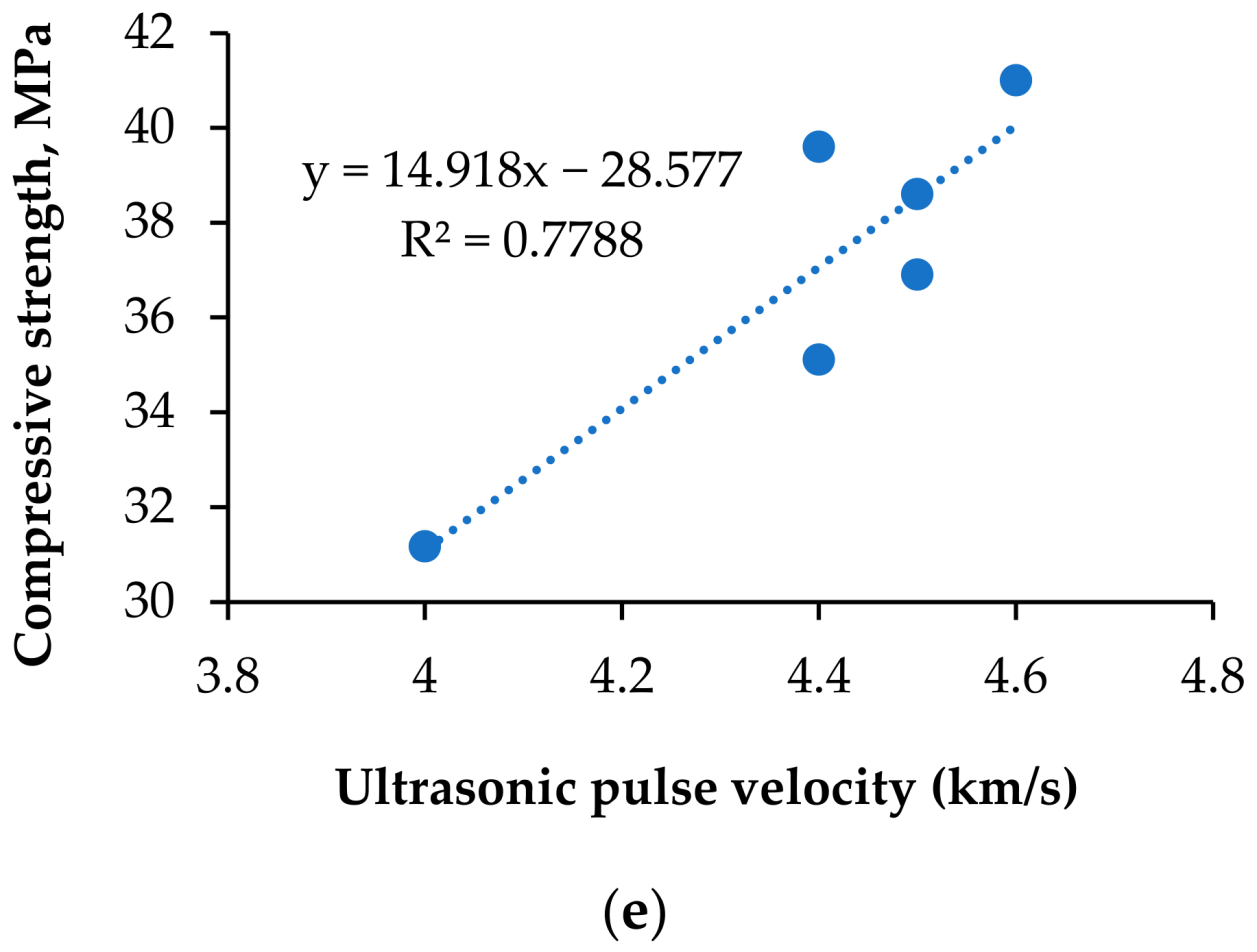
Relationship between UPV and compressive strength for CS40 group. (**a**) 0% PPF; (**b**) 0.2%PPF; (**c**) 0.4%PPF; (**d**) 0.6%PPF; (**e**) 0.8%PPF.

The transit time in µs was noted for each cube specimen at 28 days of curing. The ultrasonic pulse velocity was calculated as per the formula in Equation (1). Table 8 lists the ultrasonic pulse velocity and rebound hammer values. The pulse velocity increased to 0.6% fiber volume fraction and increased further to 0.8%. In that volume fraction, if copper slag is in lower quantity, it causes a balling effect and reduces the homogeneity and the performance of the concrete [84]. At higher volumes of copper slag content, 0.8% of PPF produces large amounts of bleeding and segregation. The fibers become clogged in separate places, leading to microcrack propagation in the concrete matrix [85]. This is the reason for the voids and non-homogenous structure of the cement concrete matrix, which delays the transit [86]. At the same time, increase in pulse velocity up to 0.6% PPF contributes to the networking texture of the PPF composite of the cement concrete matrix, and the bridging mechanism of PPF with copper slag concrete matrix. The larger the substitution, the greater the free water available. If the water–cement ratio increases, the pulse velocity decreases due to the availability of large capillary pores and microscale cracks inside the concrete [87]. Ultrasonic waves pass faster when the cement concrete matrix is filled with water than air [53]. The highest pulse velocities obtained for each replacement of copper slags were as follows: C0P3, 4700 m/s; C20P2 and C20P3, 4600 m/s; C40P2 and C40P3, 4700 m/s; C60P3, 4700 m/s; C80P3, 4600 m/s; and C100P3, 4400 m/s. The lowest pulse velocity values were obtained at 100% replacement of copper slag. Even the quality of the concrete was ascertained to be as good as the codal provisions. Figure 12 depicts the prediction of compressive strength over UPV values.

Following Equations (2) to (4) are the compressive strength equations using UPV obtained by the previous researchers. Equations (5) and (6) are the equations derived in this study. *f_c_*_’_ is the predicted compressive strength of concrete [88,89].

*f_c’_* = 20.312*UPV* − 40.959 (2)

*f_c’_* = 0.0171*UPV* − 25.478 (3)

*f_c’_* = 0.1982*UPV*^3.79^ (4)

*f_c’_* = 12.995*UPV* − 13.256 (5)

*f_c’_* = 40.336*UPV* − 142.57 (6)

#### 4.4.2. Rebound Hammer

**Figure 13:**
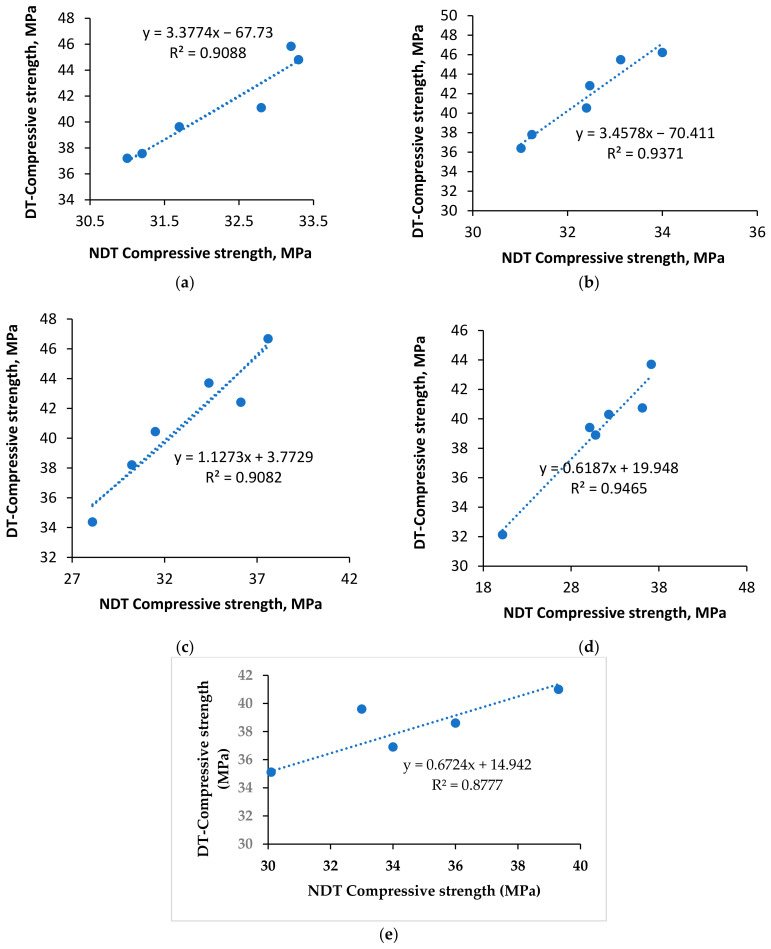
Prediction of compressive strength via rebound hammer values at each CS40% group. (**a**) 0.0%PPF; (**b**) 0.2%PPF; (**c**) 0.4%PPF; (**d**) 0.6%PPF; (**e**) 0.8%PPF.

A rebound hammer expresses the relationship between the surface hardness and the compressive strength of concrete within the limiting error. Rebound hammer observations are discarded when the variation is more than 15%. Three continuous impacts were applied perpendicular to the reference plane. Figure 13 presents the compressive strength values obtained using the rebound number. The rebound number increases as the surface resistance increases. The high specific gravity of copper slag offers more resistance to the surface indentation. CS100% shows less resistance to the hammering force. The maximum compressive strength was observed as 37.6 MPa at the C40P2 proportion. The minimum compressive strength was noted to be 20.2 MPa at the C100P3 mix. In total, 13% of results fell below 30 MPa, and all others were between 30 to 40 MPa. Except 100% copper slag replacement, all of the different mixes showed excellent resistance as per the codal provision IS 1881-Part 2 (1986) [90]. It is observed that the CS40 series showed good resistance for the surface indentation. It is concluded that the 60:40 ratio of copper slag versus sand performed well in Schmid Rebound hammer testing. The variability in our results may be connected to the curing condition and homogeneity. In copper slag–PPF concrete, the homogeneity was affected with 0.8% PPF, due to the large fiber volume. Regression equations were predicted to assess the compressive strength of concrete from the rebound hammer values. The equations are as indicated in Figure 13a–e, and the R^2^ values are nearer to 1; hence, values derived from the proposed equations fit well with the actual values.

### 4.5. Chemical Composition of Copper Slag

**Figure 14:**
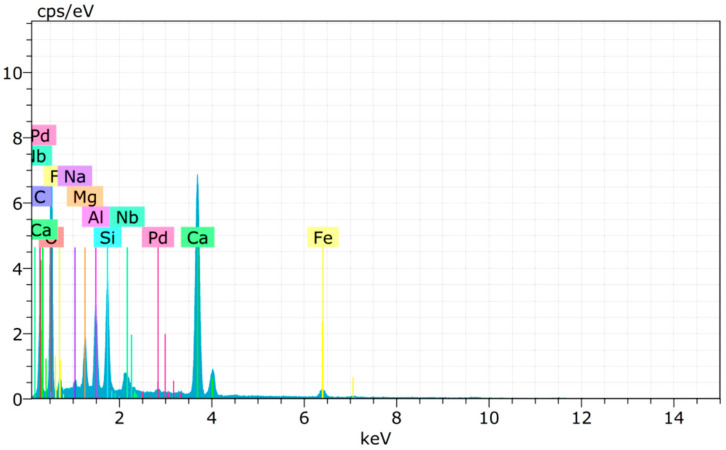
EDS pattern of ordinary Portland cement.

**Figure 15:**
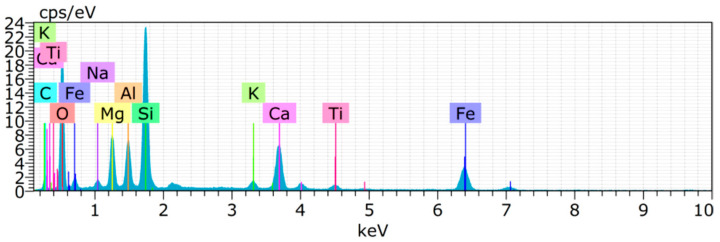
EDS pattern of copper slag.

The main components in cement and copper slag are shown in Figure 14 and Figure 15. The chemical compositions of the materials are compared in Table 3. The main components in copper slag are Fe_2_O_3_ and SiO_2_ [91]. Since the amount of calcium oxide in copper slag is relatively low, it produces a pozzolanic effect rather than exhibiting cementitious properties. Similar compounds were found in cement and copper slag, such as Ca, Si, Al, Na, Fe, Mg, etc. The high peaks from the XRD image of copper slag indicate the presence of iron silicate fines and magnetite in the crystalline form [92]. Aluminum compounds in copper slag combine with dehydrated calcium hydroxide to produce a calcium aluminate hydrate with cementitious properties [72]. Hence, it improves the dense microstructure and strength characteristics of concrete.

**Figure 16:**
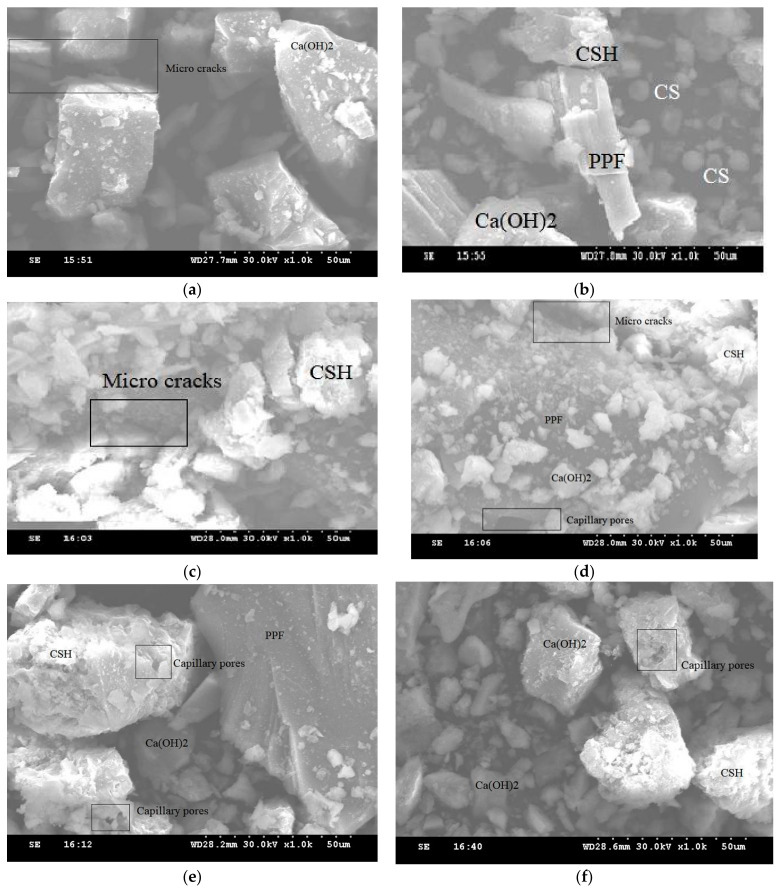
SEM images of CS–PPF concrete of CS20 group with 0.4% PPF. (**a**) CS0; (**b**) CS20; (**c**) CS40; (**d**) CS60; (**e**) CS80; (**f**) CS100.

Significant explanations were proposed regarding the SEM images of the cement aggregate matrix of the control concrete and copper slag-replaced concrete. Control concrete (Figure 16a) showed very little C–S–H gel, whereas the C20 mix (Figure 16b) showed C–S–H gel formation due to the additional silicate ions contributed by CS. In successive replacement mixes, it was evident that the presence of C–S–H gel was greater. The substitution exceeding 60% was shown with capillary pores and separation of PPF due to excess water in the concrete. C40 (Figure 16c) and C60 (Figure 16d) were identified with PPF surrounded by C–S–H gel, which was missing in the C80 (Figure 16e) proportion. The microcracks, voids, and capillary pores combined to form the networking fracture fault zones, leading to premature failure of the specimen under its service condition. At higher copper slag levels, the smooth, heavy, and glassy surface texture of copper slag induced the water to bleed to the top of the concrete, producing the weakest links in the microstructure, with available pores, voids, and microcracks [93]. In the C100 mix (Figure 16f), the PPF was not frequently observed in the microstructure images, even in 0.8% PPF. Due to lower density of PPF, it floats at the top because of excess bleeding in 100% replacement of copper slags. Since copper slag exhibits medium pozzolanic behavior, the presence of CA(Oh)_2_ is unavoidable in each mix proportion. We conclude that C–S–H gel formation increases as the replacement increases up to 60%, and further reduces due to excess water. Eventually, the strength decreases below the target level.

## 5. Conclusions

This paper extends the comparative study of copper slag concrete with polypropylene fiber (PPF) subjected to destructive and non-destructive testing. Under destructive testing of compressive strength of concrete cubes, compressive strength of mortar cubes, splitting tensile test on cylindrical specimens, and flexural tests on plain cement concrete were conducted and analyzed. Ultrasonic pulse velocity and rebound hammer tests were performed as per IS13311-Part 1-1992 for non-destructive testing. In addition, this experimental work also analyzed the microstructure of the concrete and its mechanical properties through destructive, as well as non-destructive testing. The derived conclusions are listed as follows:

- Copper slag has sharp angular lines and points that bond between aggregates. It has dimensional stability due to its high density. The density of the concrete increased within the range of 5% to 10%.
- In line with the IS: 383-1970, the gradation of 40% of copper slag replaced with sand fits with zone II. Copper slag 20%, 60%, 80%, and 100% replacements satisfy zone I criterion, which shows that the particle sizes are coarser than sand.
- Fiber-reinforced concrete alone gives equivalent or lower strength when compared to the control. From our results, copper slag addition leads to a 35% increase in the compressive strength of concrete. The application of copper slag for mortar shows better results, up to 80% replacement. Compressive strength increased to 75% was reported against the reference mix.
- The split tensile test of concrete disclosed that the maximum split tensile strength of 3.54 N/mm^2^ was perceived at the C40P4 mix, which was 31.58% greater than the control concrete. The maximum flexural strength of control concrete at 28 days for the C40P2 mix was 9.58 N/mm^2^, which is a 26.05% increase compared to control concrete.
- In cement mortar, the maximum compressive strength at 28 days was 39.11 N/mm^2^ for 20% copper slag and 0.4% PPF, which was 83% higher than normal concrete.
- Even 80% copper slag in cement mortar produced a compressive strength of 37.11 N/mm^2^ at 28 days, which was almost 75% greater than the compressive strength of the control mix.
- The concrete quality is good, and, in fact, excellent according to the ultrasonic pulse velocity test. In the rebound hammer test, 87% of results showed 30 to 40 MPa strength values, while only 13% fell below 30 MPa. The regression analysis designed to predict the compressive strength through NDT testing provided a better fit with the true values.
- The Ca(OH)_2_ crystalline structure freely available in the microstructure of control concrete is depleted in copper slag concrete, since the copper slag consumes the unreacted Ca(OH)_2_ to produce C–S–H gel.

## Figures and Tables

**Table 1:** Properties of sand, copper slag, and coarse aggregate.

Types of Aggregate	Specific Gravity	Bulk Density (g/cm^3^)	Fineness Modulus	Water Absorption (%)
Sand	2.51	1.42	3.37	1.25
Copper Slag	3.56	1.75	3.00	0.15
Coarse aggregate	2.80	1.38	6.13	0.92

**Table 2:** Chemical composition of materials used.

Chemical Composition	Cement	Copper Slag	Sand	Polypropylene Fiber
	(%)			
Oxide (O)	45.28	45.96	43.4	-
Silica (Si)	4.21	12.87	48.22	0.2
Ferrous (Fe)	3.38	9.73	0.4	-
Calcium (Ca)	26.34	8.79	-	-
Carbon (C)	12.51	8.55	7.97	99.04
Magnesium (Mg)	2.11	5.73	-	0.1
Aluminum (Al)	3.37	4.59	-	-
Sodium (Na)	0.56	1.31	-	-
Titanium (Ti)	-	1.27	-	-
Potassium (K)	-	1.19	-	-
Palladium (Pd)	0.47	-	-	0.51
Fluorine (F)	-	-	-	0.14

**Table 3:** Quantity of the material per cubic meter for all proportions.

No.	ID	Copper Slag (%)	PPF (%)	Quantity (kg/m^3^)					
				Cement	Fine Aggregate	Copper Slag	Coarse Aggregate	Water	PPF
1	Control mix	CS0	0	363	620	0	1343	148.8	0
2	C0P1		0.2	363	619	0	1343	148.8	1.82
3	C0P2		0.4	363	618	0	1343	148.8	3.64
4	C0P3		0.6	363	616	0	1343	148.8	5.46
5	C0P4		0.8	363	615	0	1343	148.8	7.28
6	C20P0	CS20	0	363	496	178	1343	148.8	0
7	C20P1		0.2	363	495	177	1343	148.8	1.82
8	C20P2		0.4	363	494	177	1343	148.8	3.64
9	C20P3		0.6	363	493	176	1343	148.8	5.46
10	C20P4		0.8	363	492	176	1343	148.8	7.28
11	C40P0	CS40	0	363	372	355	1343	148.8	0
12	C40P1		0.2	363	371	354	1343	148.8	1.82
13	C40P2		0.4	363	371	354	1343	148.8	3.64
14	C40P3		0.6	363	370	353	1343	148.8	5.46
15	C40P4		0.8	363	369	352	1343	148.8	7.28
16	C60P0	CS60	0	363	248	533	1343	148.8	0
17	C60P1		0.2	363	247	531	1343	148.8	1.82
18	C60P2		0.4	363	247	530	1343	148.8	3.64
19	C60P3		0.6	363	246	529	1343	148.8	5.46
20	C60P4		0.8	363	246	528	1343	148.8	7.28
21	C80P0	CS80	0	363	124	708	1343	148.8	0
22	C80P1		0.2	363	124	708	1343	148.8	1.82
23	C80P2		0.4	363	124	707	1343	148.8	3.64
24	C80P3		0.6	363	123	705	1343	148.8	5.46
25	C80P4		0.8	363	123	704	1343	148.8	7.28
26	C100P0	CS100	0	363	620	0	1343	148.8	0
27	C100P1		0.2	363	620	0	1343	148.8	1.82
28	C100P2		0.4	363	620	0	1343	148.8	3.64
29	C100P3		0.6	363	620	0	1343	148.8	5.46
30	C100P4		0.8	363	620	0	1343	148.8	7.28

**Table 4:** Tests conducted, test duration, and number of specimens.

No.	Name of the Testing	Duration of Testing in Days	Dimension in mm	No. of Proportions	No. of Specimens for Each Testing	Total No. of Specimens
1	Compressive strength of concrete	7, 28, 56, 90, 180	150 × 150 × 150	30	15 per proportion	450
2	Ultrasonic pulse velocity test	28	150 × 150 × 150	30	Before testing the compressive strength of concrete	
3	Rebound hammer test	28	150 × 150 × 150	30	Before testing the compressive strength of concrete	
4	SEM	28	-	6	Powder sample collected after crushing	
5	Flexural strength of concrete	28	150 × 150 × 500	30	3	90
6	Compressive strength of cement mortar	7, 28	70.6 × 70.6 × 70.6	18	6	108

**Table 5:** Velocity criterion for concrete quality.

No.	Pulse Velocity (m/sec)	Quality of Concrete
1	>4500	Excellent
2	3500 to 4500	Good
3	3000 to 3500	Medium
4	<3000	Doubtful

**Table 6:** Compressive strength of cement mortar cubes.

Mix ID	Compressive Strength of Cement Mortar at 7 Days in MPa	Compressive Strength of Cement Mortar at 28 Days in MPa	Percentage Increase in Strength at 7 Days Compared to Control Specimen	Percentage Increase in Strength at 28 Days Compared to Control Specimen
C0P0	16.00	21.30	_	_
C0P1	23.11	23.11	44.44	8.50
C0P2	19.56	26.67	22.25	25.21
C20P0	17.78	28.44	11.13	33.52
C20P1	21.33	37.33	33.31	75.26
C20P2	37.33	39.11	133.31	83.62
C40P0	17.78	23.11	11.13	8.50
C40P1	19.56	30.22	22.25	41.88
C40P2	28.44	33.78	77.75	58.59
C60P0	21.33	26.66	33.31	25.16
C60P1	24.89	28.44	55.56	33.52
C60P2	26.67	32.00	66.69	50.23
C80P0	24.89	24.88	55.56	16.81
C80P1	28.44	33.77	77.75	58.54
C80P2	30.22	37.33	88.88	75.26
C100P0	17.78	26.67	11.13	25.21
C100P1	19.56	28.44	22.25	33.52
C100P2	23.11	30.22	44.44	41.88

**Table 7:** Splitting tensile strength and flexural strength of concrete.

Mix ID	Mean Compressive Strength at 28 Days (σ_c_)	Mean Tensile Strength at 28 Days (σ_t_)	Mean Flexural Strength at 28 Days (σ_f_)	$\frac{\sigma_{t}}{\sigma_{c}}$	$\frac{\sigma_{f}}{\sigma_{c}}$	$\frac{\sigma_{t}}{\sigma_{f}}$
C0P0	37.2	2.68	7.6	0.07	0.2	0.35
C0P1	37.8	2.44	5.61	0.06	0.15	0.43
C0P2	38.2	2.68	6.23	0.07	0.16	0.43
C0P3	38.9	3.11	6.23	0.08	0.16	0.5
C0P4	39.6	3.53	5.82	0.09	0.15	0.61
C20P0	39.63	2.25	6.26	0.06	0.16	0.36
C20P1	40.53	2.54	6.82	0.06	0.17	0.37
C20P2	42.41	2.82	7.25	0.07	0.17	0.39
C20P3	40.74	3.11	6.53	0.08	0.16	0.48
C20P4	38.6	2.68	5.9	0.07	0.15	0.45
C40P0	41.1	2.42	6.35	0.06	0.15	0.38
C40P1	42.81	2.54	7.52	0.06	0.18	0.34
C40P2	46.67	2.82	7.58	0.06	0.16	0.37
C40P3	43.7	3.11	6.33	0.07	0.14	0.49
C40P4	41	3.53	5.52	0.09	0.13	0.64
C60P0	44.8	2.62	6.12	0.06	0.14	0.43
C60P1	45.48	2.82	6.89	0.06	0.15	0.41
C60P2	43.7	2.97	7.31	0.07	0.17	0.41
C60P3	40.3	3.25	6.17	0.08	0.15	0.53
C60P4	36.9	3.39	5.82	0.09	0.16	0.58
C80P0	45.84	2.6	5.93	0.06	0.13	0.44
C80P1	46.22	2.54	6.15	0.05	0.13	0.41
C80P2	40.44	2.82	6.52	0.07	0.16	0.43
C80P3	39.41	3.11	6.35	0.08	0.16	0.49
C80P4	35.11	3.39	6.57	0.1	0.19	0.52
C100P0	37.56	2.57	5.23	0.07	0.14	0.49
C100P1	36.4	2.6	5.67	0.07	0.16	0.46
C100P2	34.37	2.81	5.32	0.08	0.15	0.53
C100P3	32.13	3.21	5.71	0.1	0.18	0.56
C100P4	31.17	3.56	5.27	0.11	0.17	0.68

**Table 8:** Non-destructive testing results.

MIX ID	Ultrasonic Pulse Velocity Testing		Rebound Hammer Test
	Ultrasonic Pulse Velocity (km/s)	Quality of Concrete as per IS13311-1992 Part 1	Compressive Strength Obtained through Rebound Hammer (MPa)
C0P0	4.000	Good	31
C0P1	4.000	Good	31.25
C0P2	4.500	Good	30.23
C0P3	4.600	Excellent	30.8
C0P4	4.400	Good	31.83
C20P0	4.200	Good	31.7
C20P1	4.100	Good	32.4
C20P2	4.600	Excellent	36.13
C20P3	4.600	Excellent	36.12
C20P4	4.500	Good	33.15
C40P0	4.400	Good	32.8
C40P1	4.300	Good	32.47
C40P2	4.700	Excellent	37.6
C40P3	4.700	Excellent	37.13
C40P4	4.600	Excellent	31.95
C60P0	4.500	Good	33.3
C60P1	4.500	Good	33.12
C60P2	4.600	Excellent	34.4
C60P3	4.700	Excellent	32.3
C60P4	4.500	Good	33.9
C80P0	4.600	Excellent	33.2
C80P1	4.600	Excellent	34
C80P2	4.500	Good	31.5
C80P3	4.600	Excellent	30.12
C80P4	4.400	Good	25.07
C100P0	3.800	Good	31.2
C100P1	3.800	Good	31.02
C100P2	4.400	Good	28.1
C100P3	4.400	Good	20.2
C100P4	4.000	Good	25.63

## Data Availability

Data sharing not applicable.
